# Protocol for optogenetic control of cAMP in human telencephalic organoids using an easy-to-build Arduino-driven LED system

**DOI:** 10.1016/j.xpro.2026.104818

**Published:** 2026-09-02

**Authors:** Issei S. Shimada, Yoichi Kato

**Affiliations:** 1 Department of Cell Biology, Graduate School of Medical Sciences, Nagoya City University, Nagoya, Aichi 467-8601, Japan

**Keywords:** Cell Biology, Developmental biology, Neuroscience, Organoids, Signal Transduction

## Abstract

Temporal regulation of cyclic adenosine monophosphate (cAMP) using photoactivated adenylyl cyclase (bPAC) is critical in research; however, commercial optogenetic systems are costly. Here, we present a protocol for optogenetic control of cAMP in human telencephalic organoids using an easy-to-build Arduino-driven light-emitting diode (LED) stimulation system. We describe steps for assembling the system, writing code for LED regulation, and preparing induced pluripotent stem (iPS) cells for optogenetic experiments. We then detail procedures for optogenetic activation of cAMP to control cell-fate decisions.

For complete information on the generation and use of this protocol, please refer to Shimada et al.^1^

## Highlights

• Instructions for assembling an Arduino-driven LED controller
• Steps for generating optogenetic-tool-expressing telencephalic organoids
• Guidance on light exposure and dark-control organoid culture
• Immunostaining workflow for dorsal and ventral NSC markers

## Abstract (editor highlights)

Publisher’s note: Undertaking any experimental protocol requires adherence to local institutional guidelines for laboratory safety and ethics.

## Graphical abstract

**Figure:**
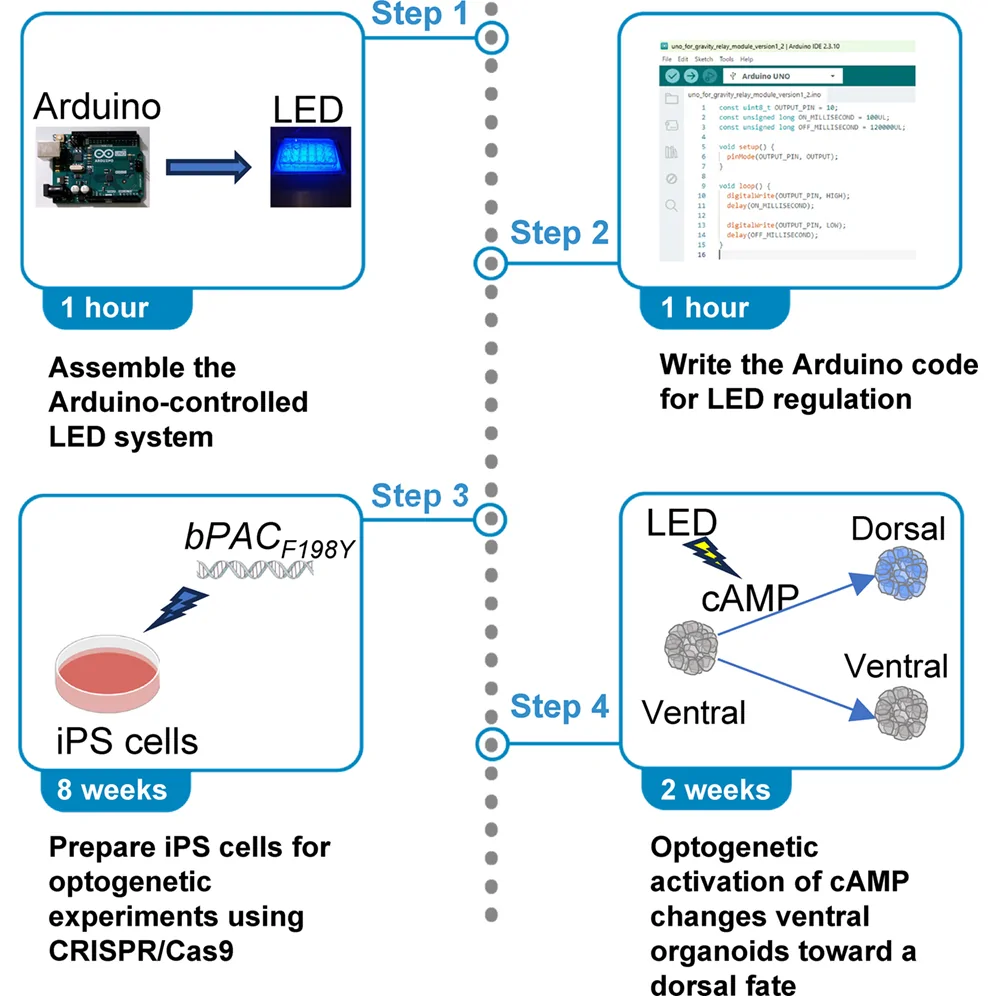

## Before you begin

Cyclic adenosine monophosphate (cAMP)^1^ is a second messenger that participates in various signaling pathways. Following activation of G protein-coupled receptors (GPCRs), G-protein α subunit (Gαs) stimulates adenylyl cyclase to produce cAMP. One critical function of cAMP is the negative regulation of Sonic hedgehog (SHH) signaling by promoting GLI family zinc finger 3 (GLI3) processing into its repressor form.^2^ SHH is a critical morphogen for brain development.^3^ During neural tube development, SHH determines the dorsal/ventral axis of neural stem/progenitor cells (NSCs). In the developing forebrain, including the telencephalon, SHH promotes ventral identities.^4^ In primary cilia, which are antenna-like organelles that protrude from the cell surface, G protein-coupled receptor 161 (GPR161)-generated cAMP activates protein kinase A (PKA). PKA then phosphorylates GLI3, which promotes its ubiquitination by β-transducin repeat-containing protein (β-TrCP) and its processing into the N-terminal GLI3 repressor.^5^ The GLI3 repressor suppresses transcription of SHH target genes. Therefore, cAMP is important for suppressing SHH signaling.

To increase cAMP levels in cells, photoactivated adenylyl cyclase (bPAC) is commonly used as an optogenetic tool.^6^ Due to the high basal activity of bPAC, a low-basal-activity mutant, bPAC_F198Y_, was developed.^7^ Here, we generate *bPAC*_*F198Y*_-expressing induced pluripotent stem (iPS) cell-derived telencephalic organoids to examine the function of cAMP during brain development. To target bPAC_F198Y_ to primary cilia, ADP-ribosylation factor-like protein 13B (Arl13b) is fused to the N terminus of bPAC_F198Y_. For application in iPS cells, we recommend generating stable *bPAC*_*F198Y*_-expressing iPS cells by targeted insertion into the adeno-associated virus integration site 1 (AAVS1) locus on chromosome 19.^8^ This protocol also includes a commonly used method for generating telencephalic organoids.

Optogenetics enables regulation of cellular processes by light stimulation. In general, optogenetic experiments require expensive commercial equipment, which may limit their use in laboratories with limited resources. The optogenetic system described here consists of four components: cells expressing a light-responsive optogenetic regulator, a 470-nm light-emitting diode (LED), an Arduino-based controller, and code that regulates the temporal pattern of illumination.

### Innovation

The high cost of commercially available optogenetic stimulation systems can interfere with research progress. This protocol reduces this financial barrier by providing an inexpensive and beginner-friendly Arduino-controlled LED system. Because no soldering is required, the device is easy to build. In addition, we provide simple Arduino code that users can copy and upload to their own Arduino. Moreover, the protocol can be used for bPAC-mediated manipulation of cAMP signaling in iPS cell-derived telencephalic organoids and can be used in various organoid research fields. This workflow makes optogenetic cAMP manipulation more accessible for organoid studies.

### Institutional permissions

Experiments using human iPS cells may require approval from the relevant institutional review board (IRB) or ethics committee, in accordance with local regulations and institutional policies. In addition, experiments involving plasmids, clustered regularly interspaced palindromic repeats (CRISPR)/Cas9, recombinant DNA, or genetically modified cells may require prior approval from the institutional biosafety committee (IBC) or other committee responsible for oversight of recombinant DNA and genetic manipulation. Users should confirm and obtain all required institutional permissions before starting the protocol.

### Software installation

**Timing: 30 min**

This section describes how to install Arduino IDE.

1. Install the latest version of Arduino integrated development environment (IDE): This protocol was tested using Arduino IDE 2.3.10. Newer compatible versions may also be used.
2. Arduino IDE can be installed on Windows 10 or later (64-bit), macOS 10.15 Catalina or later (64-bit), and Linux (64-bit).

### Purchase items for the LED controller

**Timing: 2 weeks**

This section describes how to obtain the components for the LED controller.

**Figure 1:**
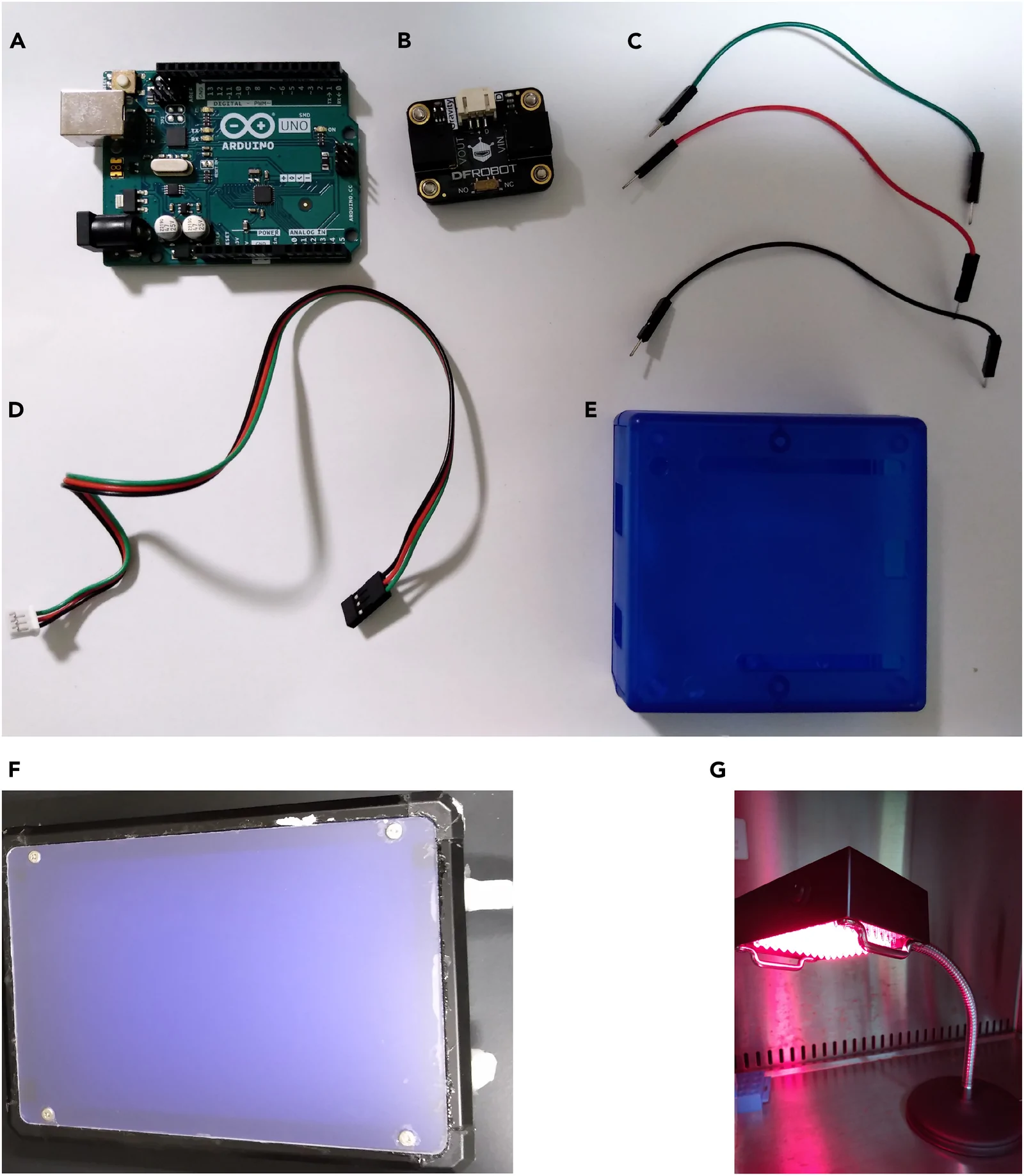
List of major components used for the Arduino and LED system (A) Arduino. (B) Gravity: Easy Relay Module. (C) Male-to-male jumper wires. (D) Digital sensor cable. (E) Arduino case. (F) 470 nm LED. (G) 660 nm LED.

3. Prepare commercially available items listed in the key resources table (Figure 1).

### Assemble the LED controller

**Timing: 1 h**

This section describes how to connect the Arduino Uno, Gravity: Easy Relay Module, power supplies, and LED illumination unit.

**Figure 2:**
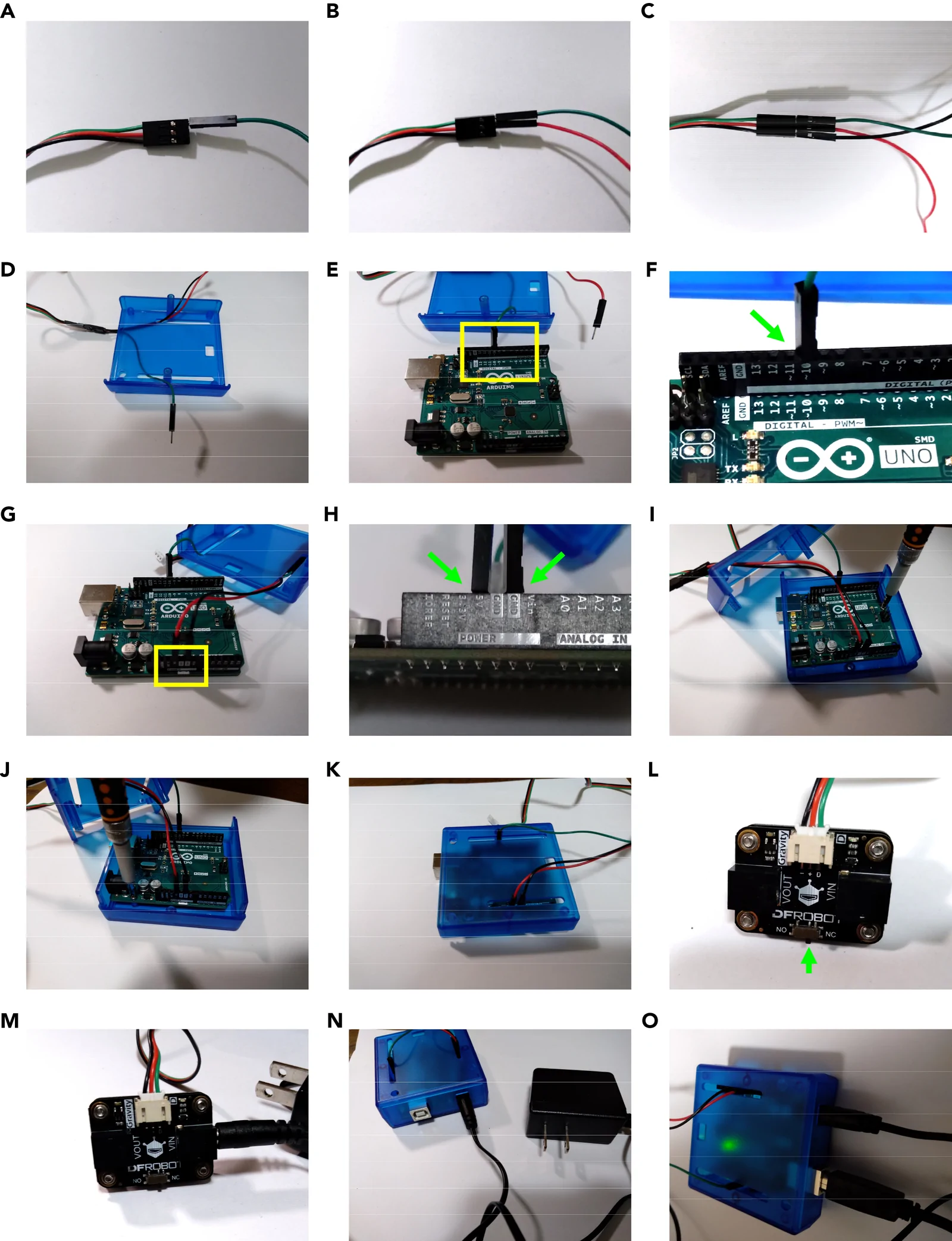
Step-by-step assembly of the Arduino controller (A–C) Connect the jumper wires to the digital sensor cable. (D) Pass the jumper wires through the gap in the case, with one wire on one side and two wires on the other side. (E and F) Connect the jumper wire to digital pin 10 on the Arduino. Panel F shows a magnified view of the yellow box in (E). The green arrow indicates the connection to digital pin 10. (G and H) Connect the jumper wires to the 5 V and GND pins. Panel H shows a magnified view of the yellow box in (G). The green arrows indicate the connections to the 5 V and GND pins. (I–K) Tighten the screws and close the case. (L) Connect the digital sensor cable to the Gravity: Easy Relay Module. Make sure that the switch is set to “NC” (green arrow). (M) Connect the 24-V power supply to the VIN connector. Connect the LED unit to the VOUT connector. (N) Connect the 5-V power supply to the Arduino. (O) Connect the USB cable to the Arduino and a computer.

***Note:*** In Figure 2L, make sure that the switch is set to “NC”.

***Note:*** Power the LEDB-SBOXHP with the 24-V DC, 0.5-A adapter and the Arduino Uno with the 5-V DC, 1.0-A adapter.

4. See Figure 2 to assemble the LED controller.
  a. Connect the three male-to-male jumper wires to the digital sensor cable (Figures 2A–2C).
  b. Pass the jumper wires through the opening in the Arduino case, with one wire on one side and two wires on the other side (Figure 2D).
  c. Connect the signal wire to digital pin 10 on the Arduino Uno (Figures 2E and 2F).
  d. Connect the remaining two wires to the 5 V and ground (GND) pins (Figures 2G and 2H).
  e. Tighten the screws and close the Arduino case (Figures 2I–2K).
  f. Connect the digital sensor cable to the Gravity: Easy Relay Module and set the relay switch to the normally closed (NC) position (Figure 2L).
  g. Connect the AC adapter providing a 24-V DC output to the VIN connector of the Gravity: Easy Relay Module, and connect the LED power cable to the VOUT connector (Figure 2M).
  h. Connect the power adapter to the Arduino controller. Connect the Arduino to a computer using a USB data cable only when uploading the program (Figures 2N and 2O).

**CRITICAL:** Do not interchange the LED and Arduino adapters.

***Note:*** Arduino recommends a 7–12-V input when the Arduino Uno is powered through its DC barrel jack. The regulated 5-V, 1.0-A adapter used in these experiments operated normally. If instability occurs, use a regulated 7–12-V, center-positive adapter compatible with the Arduino Uno barrel jack.

***Note:*** In Figure 2M, the adapter is connected to the VIN side. The LED is connected to the VOUT side. Make sure that the relay switch is set to the NC position.

**Figure 3:**
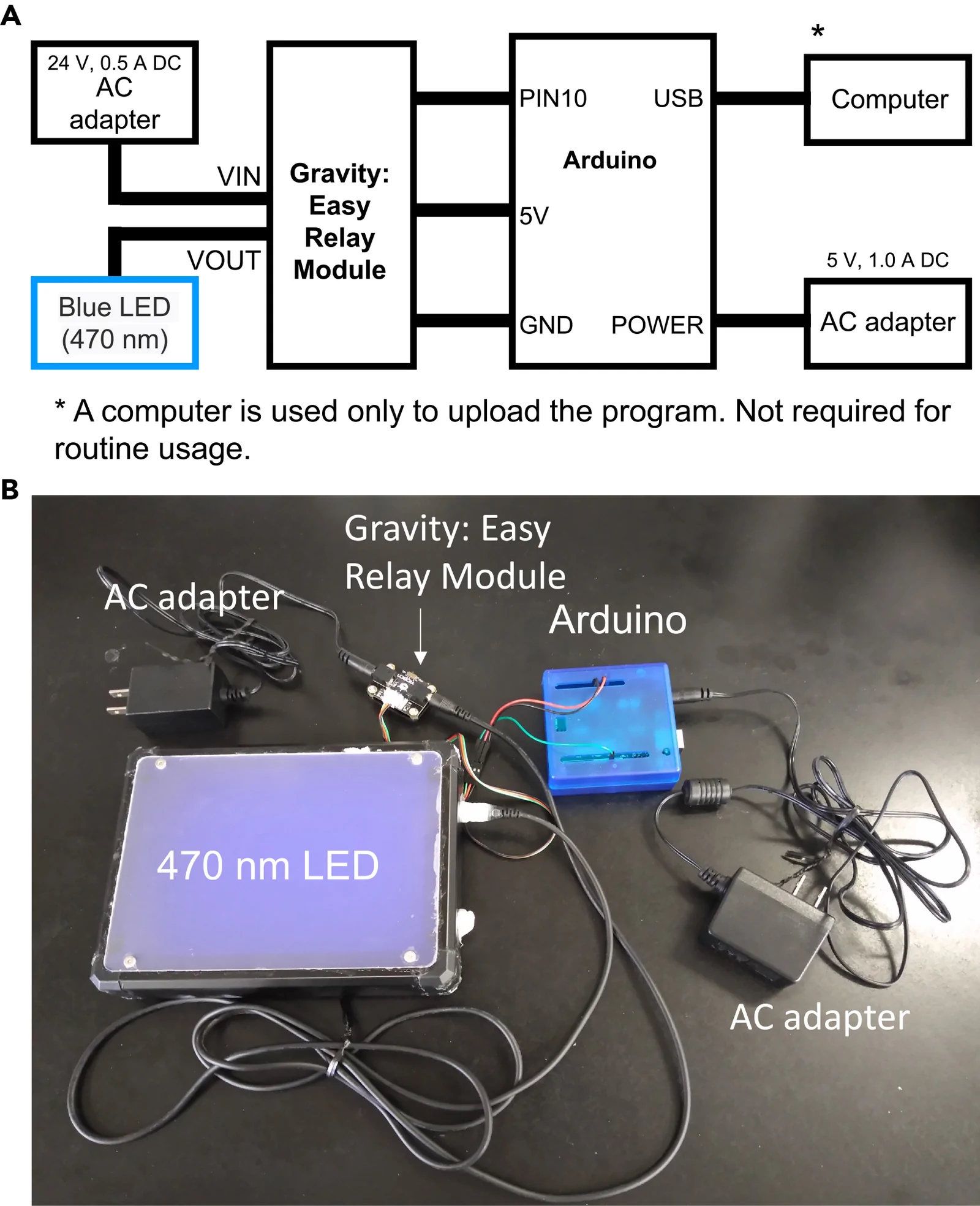
Arduino-controlled LED stimulation system The assembled (A) circuit and (B) devices.

***Note:*** A computer is connected only when the Arduino program is uploaded.

5. Confirm that the completed connections among the LED, Gravity: Easy Relay Module, and Arduino match Figure 3.

### Write the program

**Timing: 1 h**

This section describes how to create and upload the Arduino sketch used to control the illumination cycle.

**Figure 4:**
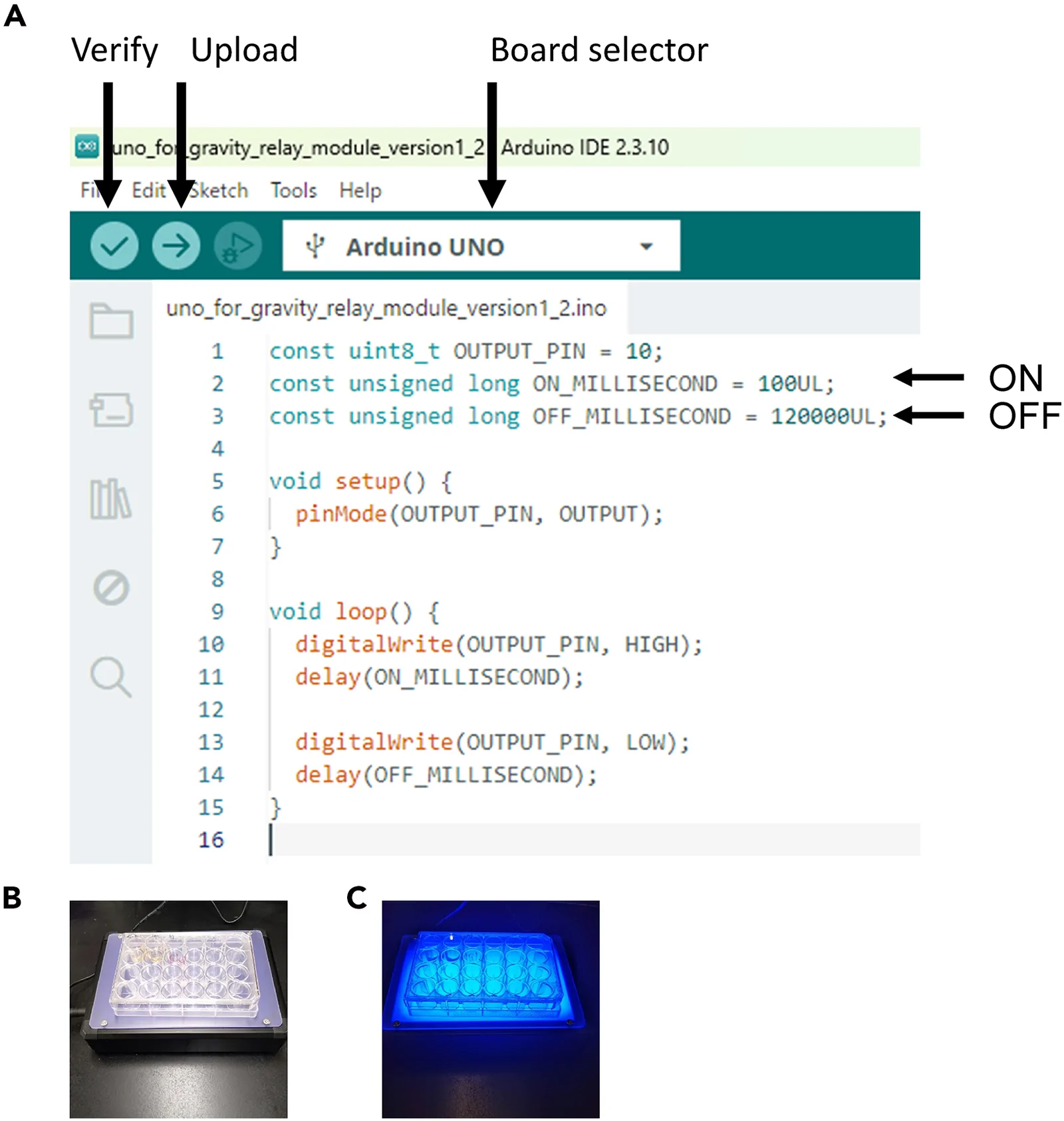
Arduino code used in the current study (A) Example code for 100 ms on and 2 min off. (B and C) LED OFF and ON states.

***Note:*** These values are in ms.

6. Open Arduino IDE and create a new sketch (File > New Sketch). Write the program as shown in Figure 4 and in the code box below for a 100 ms ON and 2 min OFF cycle.
  a. Connect the Arduino Uno to the computer with a data-capable USB cable.
  b. In the board selector, select “Arduino Uno”.
    ***Note:*** The port corresponding to the connected board is selected automatically. If the port is not selected automatically, select the connected Arduino Uno under Tools > Port.
  c. Click the “Verify” button to compile the sketch, and then click the “Upload” button (Figure 4).
    ***Note:*** Uploading takes a few seconds.
  d. To change the program, change the values of “ON_MILLISECOND” and/or “OFF_MILLISECOND”.

const uint8_t OUTPUT_PIN = 10;

const unsigned long ON_MILLISECOND = 100UL;

const unsigned long OFF_MILLISECOND = 120000UL;

void setup() {

pinMode(OUTPUT_PIN, OUTPUT);

}

void loop() {

digitalWrite(OUTPUT_PIN, HIGH);

delay(ON_MILLISECOND);

digitalWrite(OUTPUT_PIN, LOW);

delay(OFF_MILLISECOND);

}

**Pause point**: After the sketch has been uploaded successfully, disconnect the USB cable. The programmed Arduino can be used later without reconnecting it to the computer.

### Generate *GPR161* knockout iPS cells using CRISPR-Cas9

**Timing: 3*–*4 weeks**

**Timing: 1 h (step 7)**

**Timing: 1 h (step 8)**

**Timing: 3*–*4 weeks (step 9)**

In this section, we describe a protocol to generate *GPR161* KO iPS cells using CRISPR/Cas9. *GPR161* KO iPS cells will be used for generation of cells for optogenetic experiments.

***Note:*** Before you begin, culture iPS cells in a 35 mm dish with StemFit and iMatrix until they reach 60–70% confluency (about 4–7 days). iPS cells were routinely passaged every 5–7 days at 625–1,250 cells/cm^2^ and treated with 10 μM Y-27632 for the first 24 h after passaging. Please note that the optimal cell density depends on the iPS cell lines. For a 35 mm dish, use 1 ml of StemFit and 4 μl of iMatrix. Add iMatrix directly to the culture medium at 0.2 μg/cm^2^ without pre-coating the dish.

**Figure 5:**
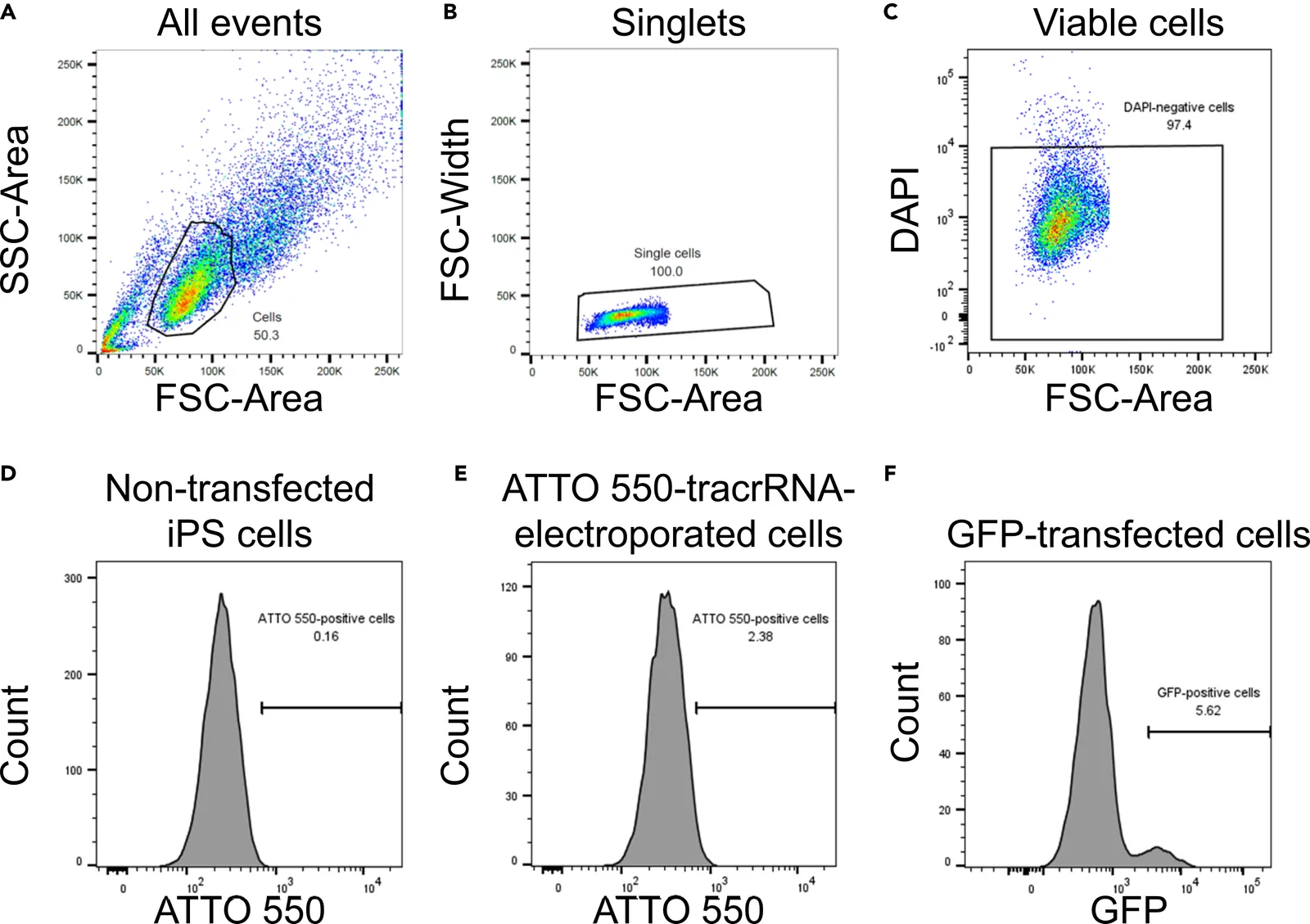
Flow cytometry plots showing the gating strategy for human iPS cells (A–C) Sequential gating of cells, singlets, and viable DAPI-negative iPS cells. (D and E) Detection of ATTO 550-labeled tracrRNA after electroporation in non-transfected iPS and ATTO 550-tracrRNA-electroporated cells, respectively. (F) Detection of GFP in *GPR161* KO cells following lipofectamine-mediated transfection with *pAAVS1-CAG-Arl13b-bPAC*_*F198Y*_*-EGFP*. Two peaks were observed, and the population with high GFP fluorescence was considered GFP-positive.
  c. Isolate individual clones using either of the following methods.
    i. Sort one cell into each well of a 96-well plate and culture for 7 days.
      ***Note:*** In our experience, the survival efficiency is about 10*–*20%, so if many clones are needed, prepare 5–10 96-well plates.
      ***Note:*** Because the volumes of iMatrix and Y-27632 required for each well are too small to pipette accurately, prepare 1 ml of StemFit containing 1 μl of Y-27632 and 1.2 μl of iMatrix for up to nine wells of a 96-well plate. Dispense 100 μl into each well before sorting. The remaining 100 μl compensates for pipetting loss. If more than nine wells are required, scale all components proportionally.
    ii. Alternatively, sort 100 cells into a 1.5 ml tube with 500 μl of StemFit supplemented with Y-27632 (10 μM), and seed the cells onto a 35 mm dish containing 1 ml of StemFit supplemented with Y-27632 and iMatrix.
      ***Note:*** This seeding density allows colonies to be manually picked after 4*–*6 days of culture. Before picking, treat the colonies with 1 ml of 0.5 mM EDTA for 1 min.
  d. During the 7-day culture period, use a microscope to identify surviving clones.
  e. Harvest approximately half of the cells from each clone with a 200 μl pipette tip for genomic DNA extraction, while retaining the remaining cells for expansion.
  f. Perform Sanger sequencing and identify the *GPR161* KO iPS clone.
    ***Note:*** The efficiency of obtaining KO lines depends on the guide RNA (gRNA). Analysis of 20 clones without identification of an edit at the target site may indicate low gRNA editing efficiency. In this case, we recommend designing a new gRNA targeting a different sequence, if possible.
  g. Expand the desired clone and freeze for future use.
    **Pause point**: The established *GPR161* KO iPS cell line can be cryopreserved at this stage.

7. Generate the CRISPR/Cas9 Ribonucleoprotein (RNP) complex.
  a. Mix 2 μl of ATTO 550-labeled tracrRNA (100 μM) and 2 μl of *GPR161* crRNA (100 μM).
    ***Note:*** The GPR161 crRNA sequence is listed in the key resources table.
  b. Incubate these RNAs at 95°C for 5 min, then allow them to cool to 20–25°C.
  c. Add 1 μl of HiFi Cas9 nuclease v3 (10 mg/ml) to the RNAs to generate the RNP complex.
  d. Incubate at 20–25°C for 30 min in the dark.
  e. During this 30 min incubation, prepare the cells for electroporation.
    ***Note:*** Mix the solution by pipetting, not by vortex.
    ***Note:*** For electroporation, use actively proliferating cells, which give higher efficiency of ATTO 550-positive cells. The cells are not separately stained with ATTO 550. ATTO 550 is covalently attached to the purchased tracrRNA. The ATTO 550-tracrRNA hybridizes with the target-specific crRNA to form the guide RNA and is incorporated into the Cas9 RNP complex. An ATTO 550-positive signal after electroporation is used as an indicator of intracellular delivery of the labeled RNP complex; it does not by itself demonstrate successful genome editing.
8. Prepare iPS cells for electroporation.
  a. Aspirate the culture medium and add 0.5 ml ethylenediaminetetraacetic acid (EDTA) solution in phosphate-buffered saline (PBS) to the dish.
    ***Note:*** If the iPS cells have been cultured for longer than seven days, incubate them with EDTA for 1 min at 37°C, then gently aspirate the EDTA and proceed to the next step.
  b. Immediately aspirate the EDTA solution and add 0.4 ml TrypLE/EDTA solution to the dish.
  c. Incubate at 37°C for 5 min in a CO_2_ incubator.
  d. Transfer the cell suspension to a 1.5 ml tube. Rinse the dish twice with 0.5 ml Dulbecco’s Modified Eagle Medium (DMEM)/F12 and collect the rinses into the same 1.5 ml tube.
    ***Note:*** Use pipetting to detach cells remaining on the dish, then transfer them. Excessive pipetting may damage the cells.
  e. Centrifuge at 190 × *g* for 3 min and collect the pellet.
  f. Resuspend it in 0.5 ml DMEM/F12 and count the number of cells.
    ***Note:*** Use a swinging-bucket centrifuge and centrifuge at 190 × g for 3 min.
  g. Transfer a volume containing 3 × 10^5^ cells to a new 1.5 ml tube. The required volume depends on the cell density. Centrifuge the tube at 190 × *g* for 3 min and aspirate the supernatant. Use the resulting cell pellet in the next step.
    ***Note:*** A 35 mm dish at approximately 60–70% confluency generally provides enough cells for two to four nucleofection experiments using 3 × 10^5^ cells per nucleofection. The remaining cells can be used for additional nucleofection experiments or discarded.
  h. Resuspend the cell pellet in 100 μl of nucleofection solution containing the *GPR161* CRISPR/Cas9 RNP complex.
  i. Transfer the solution to a nucleofection cuvette. Electroporate using Nucleofector 2b program B-016.
  j. Immediately recover the cells in 5 ml DMEM/F12 in a 15 ml tube, then centrifuge at 190 × g for 3 min and aspirate the supernatant.
  k. Resuspend the cells in 1 ml of StemFit with 1 μl of Y-27632 (10 μM final concentration), then culture them in a 35 mm dish with iMatrix.
9. Isolate single iPS cells by FACS sorting and expand the resulting clones to establish a *GPR161* KO line.
  a. The next day, collect the cells using EDTA solution followed by TrypLE/EDTA solution, and resuspend them in StemFit with Y-27632 (10 μM) and 4′,6-diamidino-2-phenylindole (DAPI; 1:500).
    ***Note:*** Because the ATTO 550 signal may decrease over time, we recommend minimal observation under a fluorescence microscope before cell collection.
  b. Isolate viable ATTO 550-positive cells using a FACSAria III sorter (Figure 5).
    i. Gate the cell population based on Forward scatter (FSC)-Area and Side scatter (SSC)-Area to exclude debris.
    ii. Select singlets using FSC-Area and FSC-Width and exclude DAPI-positive dead cells.
    iii. Define the ATTO 550-positive gate using non-transfected iPSCs stained with DAPI as a negative control and select DAPI-negative, ATTO 550-positive cells.
      ***Note:*** The commercially purchased tracrRNA was covalently labeled with ATTO 550 and was introduced into the cells during electroporation as part of the Cas9 RNP complex. ATTO 550-positive cells were isolated to enrich for cells that had received the genome-editing reagents.
      ***Note:*** About 1*–*10% of cells are ATTO 550-positive.
      ***Note:*** Prepare a tube containing non-transfected iPS cells stained with DAPI as a negative control for ATTO 550.

### Generate cells for optogenetics

**Timing: 3–4 weeks**

In this section, we describe how to insert a *bPAC*_*F198Y*_-*enhanced green fluorescent protein* (*EGFP)* fusion construct into the AAVS1 locus of iPS cells using CRISPR/Cas9. *GPR161* KO iPS cells will be used for generation of cells for optogenetics. bPAC_F198Y_ is used instead of bPAC because it has lower activity in the dark. We also use primary cilium-targeted bPAC_F198Y_-EGFP and cytoplasm-targeted bPAC_F198Y_-EGFP to analyze the effects of cAMP production in primary cilia and the cytoplasm.

10. Generate *bPAC*_*F198Y*_*-EGFP*-expressing *GPR161* KO iPS cells for optogenetics
  a. Seed 6,000 *GPR161* KO iPS cells in a 24-well plate.
  b. The next day, transfect the cells with the plasmids using Lipofectamine Stem Transfection Reagent.
    ***Note:*** For each well of a 24-well plate, dilute 1 μl of Lipofectamine Stem Transfection Reagent in 25 μl of Opti-MEM™ I Reduced Serum Medium. Separately, dilute 250 ng of *pAAVS1-CAG-bPAC*_*F198Y*_*-EGFP* or *pAAVS1-CAG-Arl13b-bPAC*_*F198Y*_*-EGFP,* together with 250 ng of *pXAT2*, in 25 μl of Opti-MEM™ I Reduced Serum Medium. Combine the two solutions, vortex for 2–3 s, and incubate at 20–25°C for 10 min. Add the resulting transfection mixture dropwise to the cells in the 24-well plate. The pAAVS1 vector and pXAT2 are used to target the AAVS1 locus on chromosome 19 by CRISPR/Cas9.
  c. The next day, visualize GFP-positive cells under a fluorescence microscope.
    ***Note:*** The number of GFP-positive cells will decrease over time, since only a subset of cells will have genomic integration. Transient GFP expression from non-integrated plasmid DNA decreases over time. Cells with stable genomic integration of the expression cassette are expected to retain GFP expression after 2 weeks.
  d. Culture the cells until confluency (4–7 days).
  e. Sort GFP-positive iPS cells using a FACSAria III cell sorter according to the gating strategy shown in Figures 5A–5C and 5F.
    ***Note:*** Sort one GFP-positive cell into each well of a 96-well plate and culture.
    ***Note:*** Before sorting, prepare the 96-well plate with StemFit, Y-27632, and iMatrix as described in the Note following step 9c(i).
  f. After confirming stable GFP expression, freeze the cells for future use.
    ***Note:*** It is important to confirm GFP expression two weeks after the lipofectamine transfection before future use.
    **Pause point**: The iPS cells can be cryopreserved at this stage.

## Key resources table

**Table:** 

REAGENT or RESOURCE	SOURCE	IDENTIFIER
**Antibodies**		

ARL13B (1:500 dilution)	Proteintech	Cat. # 17711-1-AP, RRID:AB_2060867
GFP (B-2) (1:500 dilution)	Santa Cruz	Cat. # sc-9996, RRID:AB_627695
NKX2.1 (TTF-1; 8G7G3/1) (1:500 dilution)	Diagnostic BioSystems	Cat. # MOB285-01, RRID:AB_3730160
PAX6 (1:500 dilution)	BioLegend	Cat. # 901301, RRID:AB_2565003
Goat anti-Rabbit Secondary Antibody, Alexa Fluor 594 (1:500 dilution)	Invitrogen	Cat. # A-11037, RRID:AB_2534095
Goat anti-Rabbit Secondary Antibody, Alexa Fluor 647 (1:500 dilution)	Invitrogen	Cat. # A-21244, RRID:AB_2535812
Goat anti-Mouse IgG1 Secondary Antibody, Alexa Fluor 555 (1:500 dilution)	Invitrogen	Cat. # A-21127, RRID:AB_141596
Goat anti-Mouse IgG2a Secondary Antibody, Alexa Fluor 488 (1:500 dilution)	Invitrogen	Cat. # A-21131, RRID:AB_141618

**Chemicals, peptides, and recombinant proteins**		

TrypLE Express Enzyme	Gibco	Cat. # 12604021
Disodium EDTA dihydrate	Sigma-Aldrich	Cat. # E5134
StemFit® AK02N	Ajinomoto	Cat. # RCAK02N
Y-27632 Dihydrochloride	Chemscene	Cat. # CS-0878
iMatrix-511 silk	Takara Bio	Cat. # T311
Stem Cell Banker	Takara Bio	Cat. # CB045
Alt-R tracrRNA with ATTO 550	Integrated DNA Technologies	Cat. # 1075927
Alt-R S.p.HiFi Cas9 Nuclease V3	Integrated DNA Technologies	Cat. # 1081060
Electroporation Cuvette, 0.2 cm	Bio-Rad	Cat. # 1652086
DAPI (10 mg/ml)	Sigma-Aldrich	Cat. # D8417
DMEM/F12	Invitrogen	Cat. # 11330-032
ATP	Sigma-Aldrich	Cat. # 10519979001
Magnesium chloride hexahydrate	Sigma-Aldrich	Cat. # 19-0190-5
Potassium phosphate monobasic	Sigma-Aldrich	Cat. # P9791
D-(+)-Glucose	Nacalai Tesque	Cat. # 16806
Sodium hydroxide	Sigma-Aldrich	Cat. # 795429
Sodium bicarbonate	FUJIFILM Wako Pure Chemical	Cat. # 191-01305
96-well round bottom ultra-low attachment microplate	Corning	Cat. # 7007
Nunclon Sphera 96-Well, Nunclon Sphera-Treated, U-Shaped-Bottom Microplate	Thermo Fisher Scientific	Cat. # 174925
KnockOut Serum Replacement	Thermo Fisher Scientific	Cat. # 10828010
MEM Non-essential amino acids solution (MEM-NEAA; 100X)	Gibco	Cat. # 11140050
LDN-193189 (hydrochloride)	Cayman Chemical	Cat. # 19396
SB-431542 (hydrate)	Cayman Chemical	Cat. # 13031
XAV939	Cayman Chemical	Cat. # 13596
2-mercaptoethanol	FUJIFILM Wako Pure Chemical	Cat. # 133-14571
200 mM L-Glutamine	Nacalai Tesque	Cat. # 16948-04
Penicillin-Streptomycin Mixed Solution	Nacalai Tesque	Cat. # 26253-84
MACS Neuro Medium	Miltenyi Biotec	Cat. # 130-093-570
Insulin	Nacalai Tesque	Cat. # 12878-86
N-2 MAX Media Supplement	R&D Systems	Cat. # AR009
NeuroBrew-21 Supplement without Vitamin A	Miltenyi Biotec	Cat. # 130-097-263
NeuroBrew-21 Supplement	Miltenyi Biotec	Cat. # 130-093-566
L-Ascorbic acid	Sigma-Aldrich	Cat. # A92902
Paraformaldehyde	FUJIFILM Wako Pure Chemical	Cat. # 162-16065
Tissue-Tek OCT Compound	Sakura Finetek Japan	Cat. # 4583
Sucrose	Nacalai Tesque	Cat. # 09589-05
Triton X-100	Sigma-Aldrich	Cat. # 30-5140-5
Fluoromount-G	SouthernBiotech	Cat. # 0100-01
Normal Serum Block (Goat)	BioLegend	Cat. # 927503
Q5 High-Fidelity DNA Polymerase	New England Biolabs	Cat. # M0491L
Disposable Base Molds 15 mm X 15 mm	TED PELLA	Cat. # 27147-2
Opti-MEM™ I Reduced Serum Medium	Gibco	Cat. # 31985-062
Fisherbrand™ Superfrost™ Plus Microscope Slides	Fisher Scientific	Cat. # 12-550-15
Micro slide glass Frontier	Matsunami Glass	Cat. # FRC-01
NEO Coverslips	Matsunami Glass	Cat. # C024601

**Critical commercial assays**		

Lipofectamine Stem Transfection Reagent	Thermo Fisher Scientific	Cat. # STEM00001
DNeasy Blood and Tissue Kits for DNA Isolation	Qiagen	Cat. # 69504

**Experimental models: Cell lines**		

Human 201B7 iPS cells	Riken	201B7
Human 201B7 iPS cells *GPR161* KO2	Shimada et al.,^1^	N/A
*Cyto-bPAC*_*F198Y*_*-EGFP* 201B7 *GPR161* KO2 iPS cells	Shimada et al.,^1^	N/A
*Cilia-bPAC*_*F198Y*_*-EGFP* 201B7 *GPR161* KO2 iPS cells	Shimada et al.,^1^	N/A

**Oligonucleotides**		

GPR161 crRNA-2 (Design ID: Hs.Cas9.GPR161.1.AE)	Integrated DNA Technologies	GATCTTCATGGGGTACACCA
sgGpr161-F	Integrated DNA Technologies	CCCTCCATCCCTTTTCTAGC
sgGpr161-R	Integrated DNA Technologies	CTCTGAGCATCCTCCTCCAC
GPR161ACseq primer-R1	Integrated DNA Technologies	CCATAGCACACCAGCATGAC

**Recombinant DNA**		

pXAT2	Oceguera-Yanez et al.^8^	Addgene: #80494
pAAVS1-CAG-bPAC_F198Y_-EGFP	Shimada et al.,^1^	N/A
pAAVS1-CAG-Arl13b-bPAC_F198Y_-EGFP	Shimada et al.,^1^	N/A

**Software and algorithms**		

Fiji (ImageJ) (Version 1.54f)	RRID:SCR_002285	https://imagej.net/software/fiji/downloads
Olympus Fluoview (Version 2.6)	RRID:SCR_020339	https://www.olympus-lifescience.com/en/laser-scanning/fv3000/
BD FACSDiva (Version 9.4)	RRID:SCR_001456	https://www.bdbiosciences.com/en-us
FlowJo (Version 10.8.1)	RRID:SCR_008520	https://www.flowjo.com/
Arduino IDE 2.3.10	RRID:SCR_024884	https://www.arduino.cc/en/software
Arduino code for temporal control of LED illumination	This paper	https://zenodo.org/records/21673121

**Other**		

35 mm dish	Corning	Cat. # 430165
6-well plate	TPP	Cat. # 92406
12-well plate	TPP	Cat. # 92412
24-well plate	TPP	Cat. # 92124
96-well plate	TPP	Cat. # 92696
Arduino Uno SMD R3	Arduino	Cat. # A000073
Gravity: Easy Relay Module	DFRobot	Cat. # DFR0643
Male-to-male jumper wire	Digikey	Cat. # 1528-1967-ND
Digital Sensor Cable for Arduino	DFRobot	Cat. # FIT0756
Arduino case	Hammond Manufacturing	Cat. # 1593HAMARTBU
470-nm LED device	Optocode	Cat. # LEDB-SBOXHP
660-nm LED stand light	Optocode	Cat. # LED660-100STND
Fluoview FV3000 Confocal microscope	Olympus	N/A
BD FACSAria III	BD Biosciences	N/A
Orbital shaker	TAITEC	Cat. # CS-LR
Nucleofector 2b	Lonza	Cat. # AAB-1001
Cryostat	Leica	CM1860UV
CO_2_ incubator	PHCbi	Cat. # MCO-170AIC-PJ
USB Type-A to Type-B data cable	Sanwa Supply	Cat. # KU-R1
AC adapter for LEDB-SBOXHP	Li Tone Electronics	LTE10UW-S4-BSA1; 24 V DC, 0.5 A; center-positive
AC adapter for the Arduino controller	Linkman	ATS005T-W050U; 5 V DC, 1.0 A; center-positive

## Materials and equipment

**Table:** Induction medium (final volume: 10 ml)

Reagent	Stock concentration	Final concentration	Amount
DMEM/F12	1×	-	7.67 mL
KnockOut Serum Replacement	100%	20%	2 mL
MEM-NEAA	100×	1×	100 μL
L-glutamine	100×	1×	100 μL
Pen/Strep	100×	1×	100 μL
2-mercaptoethanol	1% (144 mM)	0.0007% (100 μM)	7 μL
LDN-193189	2 mM	100 nM	0.5 μL
SB-431542	10 mM	10 μM	10 μL
XAV939	2 mM	2 μM	10 μL

**Storage**: Store at 4°C for up to 1 week.

**Table:** Cortical organoid medium 1 (final volume: 50 mL)

Reagent	Stock concentration	Final concentration	Amount
DMEM/F12	100%	50%	23.86 mL
MACS Neuro medium	100%	50%	23.86 mL
N2 MAX supplement	100×	0.5×	250 μL
L-glutamine	100×	1×	500 μL
MEM-NEAA	100×	1×	500 μL
Insulin	9.5–11.5 mg/ml	∼2.5 μg/ml	12.5 μL
2-mercaptoethanol	1% (144 mM)	0.00035% (50 μM)	17.5 μL
Pen/Strep	100×	1×	500 μL
NeuroBrew-21 Supplement without vitamin A	50×	0.5×	500 μL

**Storage**: Store at 4°C for up to 1 month.

**Table:** Cortical organoid medium 2 (final volume: 50 ml)

Reagent	Stock concentration	Final concentration	Amount
DMEM/F12	100%	50%	23.86 mL
MACS Neuro medium	100%	50%	23.86 mL
N2 MAX supplement	100×	0.5×	250 μL
L-glutamine	100×	1×	500 μL
MEM-NEAA	100×	1×	500 μL
Insulin	9.5–11.5 mg/ml	∼2.5 μg/ml	12.5 μL
2-mercaptoethanol	1% (144 mM)	0.00035% (50 μM)	17.5 μL
Pen/Strep	100×	1×	500 μL
Ascorbic acid	1 M	200 μM	10 μL
NeuroBrew-21 Supplement	50×	0.5×	500 μL

**Storage**: Store at 4°C for up to 1 month.

**Table:** Solution A

Reagent	Stock concentration	Amount
ATP	330 mM	2 g
MgCl_2_-6H_2_O	590.3 mM	1.2 g
H_2_O	—	Bring to 10 ml

**Storage**: Store at −20°C for up to 1 year.

**Table:** Solution B

Reagent	Stock concentration	Amount
KH_2_PO_4_	88.2 mM	6 g
NaHCO_3_	14.3 mM	0.6 g
Glucose	2.22 mM	0.2 g
NaOH	—	Adjust to pH 7.4
H_2_O	—	Bring to 500 ml

**Storage**: Store at −20°C for up to 1 year.

**Table:** Nucleofection solution (final volume: 500 μl)

Reagent	Amount
Solution A	10 μL
Solution B	490 μL

**Storage**: Prepare fresh and use immediately.

**Table:** EDTA stock solution (final volume: 50 mL; final concentration: 0.5 M; pH 8)

Reagent	Amount
Disodium EDTA-2H_2_O	9.31 g
NaOH	Adjust to pH 8.0
H_2_O	Bring to 50 ml

**Storage**: Store at 4°C for up to 12 months.

**Table:** EDTA solution (final volume: 50 mL; final concentration: 0.5 mM)

Reagent	Amount
0.5 M EDTA	50 μL
PBS (pH 7.4)	49.95 mL

**Storage**: Store at 4°C for up to 6 months.

**Table:** TrypLE/EDTA solution (final volume: 50 ml)

Reagent	Amount
TrypLE	25 mL
0.5 mM EDTA solution	25 mL

**Storage**: Store at 4°C for up to 6 months.

## Step-by-step method details

### Generate telencephalic organoids from iPS cells with the LED

**Timing: 2 weeks**

In this section, we describe how to generate telencephalic organoids from the engineered iPS cells using programmed LED exposure. Two CO_2_ incubators are required: one for the light-exposure plate and one for the dark-control plate. Other cultures may share the incubator as long as opening the incubator does not disrupt the programmed light-exposure or dark-control conditions.

***Note:*** The irradiance was approximately 6.0 mW/cm^2^ at approximately 5 mm from the illumination surface, measured approximately 1 min after the LED was turned on. This value is included as reference information provided by the manufacturer.

***Note:*** A 660-nm LED lamp should be installed in the tissue-culture hood and used during cell handling in a dark room.

1. Culture iPS cells in one to two wells of a 12-well plate until they reach 60–70% confluency.

***Note:*** One to two wells generally provide enough cells for one 96-well plate, although the yield depends on the cell line and confluency.

2. Dissociate the iPS cells and count the number of cells.
3. Prepare the required number of cells at 3,000 cells per well.
  a. For 12 wells of a 96-well plate, prepare 39,000 cells in 1.3 ml of induction medium, corresponding to 36,000 cells for 12 wells plus an excess equivalent to one additional well.
  b. For a full 96-well plate, prepare at least 300,000 cells in 10 ml of induction medium.

***Note:*** Always prepare a small excess volume to compensate for pipetting loss.

4. Centrifuge the cell suspension at 190 × g for 3 min and aspirate the supernatant.
5. Resuspend the cells at 3,000 cells per 100 μl in induction medium containing 50 μM Y-27632, and dispense 100 μl into each well of a U-bottom ultra-low binding 96-well plate.

***Note:*** Place only the LED illumination unit inside the incubator, and keep the Arduino controller and power supply outside. Route the LED cable through an incubator access port, if available. If no access port is available, pass the cable through the gap between the incubator door and frame without pinching or sharply bending it. Close the door and confirm that the remaining gap is minimal. If necessary, cover any small residual gap around the cable with Parafilm.

**CRITICAL:** After routing the LED cable, confirm that the incubator temperature and CO_2_ concentration remain stable.

6. Establish the light-exposure and dark-control conditions.
  a. Place the 96-well plate on the LED and turn on the switch for the light-exposure condition.
  b. Place another 96-well plate into a separate CO_2_ incubator for the dark-control condition.

**CRITICAL:** From step 6 onward, handle both the light-exposure and dark-control cultures under a 660-nm LED light.

**CRITICAL:** Make sure the room is dark, and do not open the light-exposed or dark-control CO_2_ incubator when the room lights are on.

7. Change half of the medium every 2–3 days until day 6.

***Note:*** Y-27632 is used only for the first two days; therefore, Y-27632 is not required at this stage.

***Note:*** Using a 200 μl tip, gently aspirate 50 μl of medium from the top surface. Healthy organoids sink to the bottom, so aspirating from the top helps prevent accidental removal of organoids.

***Note:*** Use a dark blue sheet or object on the clean bench to help visualize the organoids. Because the organoids are white or transparent, a dark blue background makes them easier to see.

***Note:*** If you do not see the aggregates at the bottom on day 1, it is possible that Y-27632 was not added on day 0.

8. On day 6, aspirate all of the medium and then add 100 μl of cortical organoid medium 1.

***Note:*** Completely aspirating all the medium may be difficult at first. In that case, aspirate about 90% of the medium and add 100 μl of cortical organoid medium 1. Then aspirate about 90% of the medium again and add another 100 μl of cortical organoid medium 1. This stepwise dilution method makes the medium change easier.

9. Change half of the medium every 2–3 days until day 14.

***Note:*** For organoid culture beyond day 14, transfer the organoids to a 6-well plate. Replace the medium with 1.5 ml of cortical organoid medium 2, and culture them on an orbital shaker, such as Taitec CS-LR or equivalent, at 70 rpm. Replace half of the medium every 1–4 days. Because the orbital shaker platform is larger than the LED unit, the LED unit can be placed on the shaker and the culture plate positioned on top of it. Make sure that the cable has sufficient slack and does not become tangled, caught, or disconnected during shaking.

### Fix and stain the organoids

**Timing: 1 week**

This section describes how to fix, cryoprotect, cryosection, stain, and image the telencephalic organoids.

10. Transfer the organoids to a new 1.5 ml tube and fix them with 700 μl of 4% paraformaldehyde in PBS at 4°C for 3–4 h.

***Note:*** To collect organoids, cut the end of a pipette tip to widen the opening.

11. Discard the paraformaldehyde and add 700 μl of 30% sucrose in PBS and incubate at 4°C for 12–16 h.
12. Transfer the organoids to a disposable base mold and aspirate the remaining sucrose. Add OCT compound and freeze the OCT-embedded organoids at −80°C.

***Note:*** Position the organoids near one edge of the mold. This makes it easier to locate the organoid when sectioning.

**Pause point**: Store the OCT-embedded organoids at −80°C until sectioning.

13. Cut 16-μm frozen sections using a cryostat and transfer the sections onto adhesive slides to reduce detachment of OCT-embedded tissue during staining.

**Pause point**: If not used immediately, store the sections at −20°C for up to 1 year.

14. Block and stain the sections.
  a. Wash the sections in PBS to dissolve OCT compound, then block at 20–25°C for 1 h in blocking buffer containing 3% normal goat serum and 0.3% Triton X-100 in PBS.
  b. Incubate with the primary antibodies diluted in the blocking buffer containing 3% normal goat serum and 0.3% Triton X-100 in PBS at 4°C for 12–16 h.
  c. Wash with PBS and incubate with secondary antibodies and DAPI (1:500) diluted in the blocking buffer containing 3% normal goat serum and 0.3% Triton X-100 in PBS for 1 h at 20–25°C.
  d. Mount the sections with coverslips using Fluoromount-G.
15. Acquire images using a microscope.

## Expected outcomes

The LED turns on and off according to the programmed settings. In this protocol, we used a 100 ms ON and 2 min OFF cycle. The duration of the cycle can be changed depending on the experimental design. Figure 3 shows the assembled devices, and Figures 4B and 4C show programmed LED OFF and ON states, respectively.

**Figure 6:**
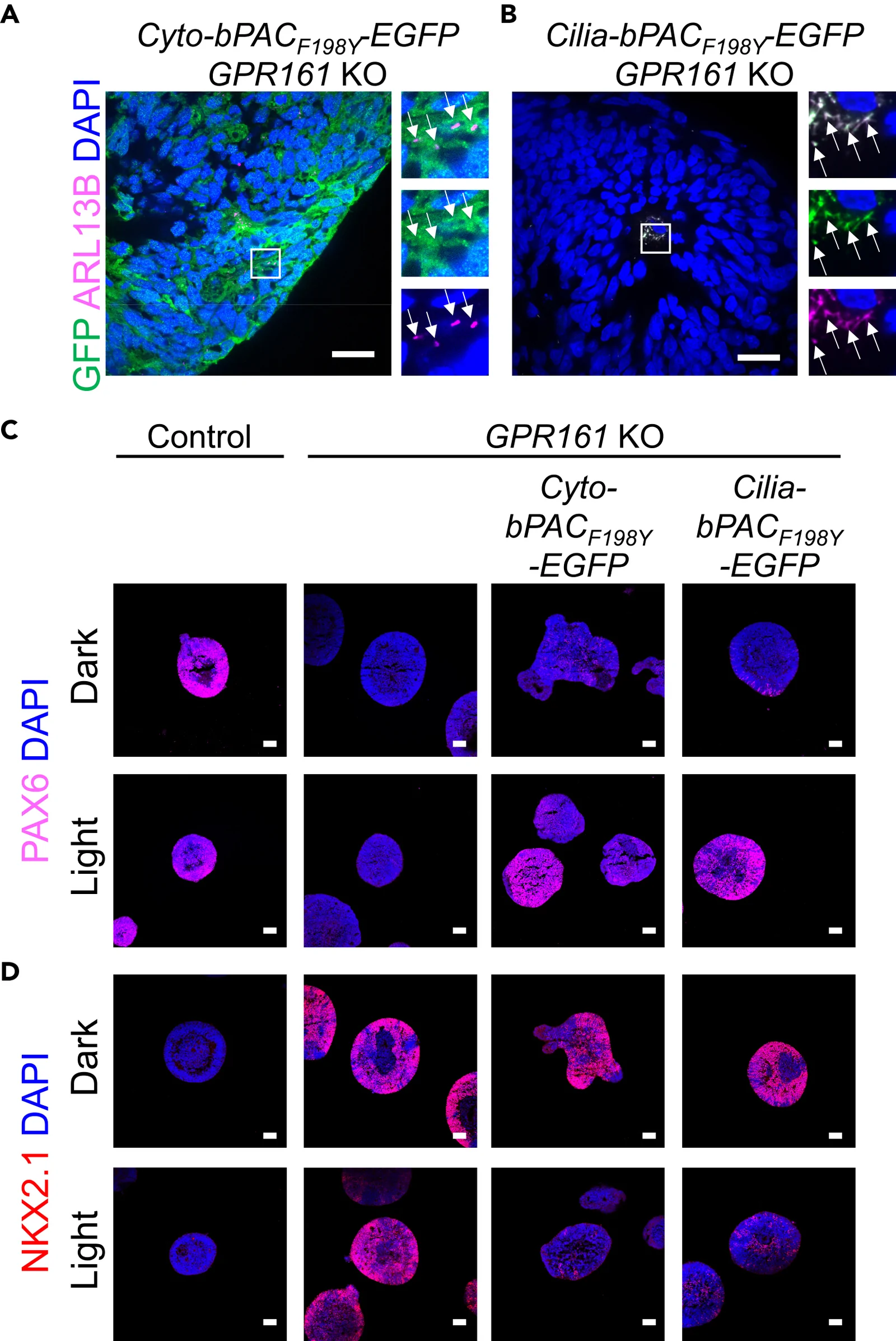
Programmed light exposure regulates dorsal/ventral patterning of *GPR161* KO organoids (A and B) GFP signals in the ventricular zone of *cyto-bPAC*_*F198Y*_*-EGFP*- and *cilia-bPAC*_*F198Y*_-*EGFP-*expressing *GPR161* KO organoids. *Cilia*-bPAC_*F198Y*_-EGFP was observed in ARL13B-positive primary cilia. White boxed regions are enlarged on the right, showing separate channels and merged images. White arrows indicate primary cilia. (C and D) Representative images of (C) PAX6-positive or (D) NKX2.1-positive NSCs in the ventricular zones of telencephalic organoids. Scale bars: (A and B) 20 μm and (C and D) 100 μm.

Using *GPR161* KO iPS cells expressing either *cytoplasmic* (*cyto*)-*bPAC*_*F198Y*_*-EGFP* or *cilia*-*bPAC*_*F198Y*_*-EGFP*, we generated telencephalic organoids (Figures 6A and 6B). Immunohistochemistry showed GFP localization in the cytoplasm of *cytoplasmic*-*bPAC*_*F198Y*_*-EGFP-GPR161* KO organoids or in the cilia of *cilia*-*bPAC*_*F198Y*_*-EGFP-GPR161* KO organoids (Figures 6A and 6B). Without light exposure, *GPR161* KO organoids showed reduced expression of the dorsal NSC marker, paired box 6 (PAX6), and increased ectopic expression of the ventral NSC marker NK2 homeobox 1 (NKX2.1) in the ventricular zone of the organoids (Figures 6C and 6D). Since *GPR161* deletion reduces cAMP levels, we increased cAMP by activating either *cyto*- or *cilia*-targeted *bPAC*_*F198Y*_. After two weeks of light exposure, consistent with the quantitative findings reported by Shimada et al*.*,^1^ representative images showed decreased NKX2.1 staining and increased PAX6 staining under both *cyto*-*bPAC*_*F198Y*_ and *cilia*-*bPAC*_*F198Y*_ conditions. These data are consistent with successful optogenetic elevation of cAMP signaling.

## Limitations

One of the limitations of the programmed LED system is that it relies on the internal clock of the Arduino. Compared with highly accurate reference clocks, the Arduino clock has some variability. Therefore, over a 2-week period, there may be slight drift in the exact timing of light cycles relative to absolute time and, consequently, slight variation in the number of cycles delivered within a fixed interval. Nevertheless, the current device was sufficient to affect biological systems in the organoids, indicating that it may serve as a first step toward broader use of optogenetics in the life sciences.

A limitation of this system is its scalability. Large-batch experiments may require multiple LED illumination units, and scaling long-term cultures to multiple plates may also require multiple orbital shakers.

## Troubleshooting

### Problem 1

The Arduino IDE shows an error and the LED program does not work.

Related to step 6 in “Write the program.”

### Potential solution

Make sure the code has no typos.

Make sure that the code was uploaded successfully.

Check the wire connections among all components.

Check the version of the Arduino IDE.

### Problem 2

The LED does not light up.

Related to steps 4–6 under “before you begin”.

### Potential solution

Check the connections among the LED unit, Gravity: Easy Relay Module, 24-V adapter, Arduino, and jumper wires. High temperature and humidity may damage the LED illumination unit.

### Problem 3: The organoids do not form

Related to steps 1–4 in “Generate telencephalic organoids from iPS cells with the LED.”

### Potential solution

Confirm that the induction medium is fresh.

Confirm that Y-27632 was included.

Some iPS cell lines do not efficiently generate telencephalic organoids. Use 201B7 iPS cells or another iPS cell line that has been shown to generate telencephalic organoids.

### Problem 4: Organoids in the light-exposed condition look unhealthy after 2 weeks

Related to steps 6–9 in “Generate telencephalic organoids from iPS cells with the LED.”

### Potential solution

Prolonged LED exposure may increase the culture temperature or otherwise stress the organoids. Confirm that the incubator temperature remains stable during illumination, and reduce the illumination duration or frequency if necessary. To assess possible cell stress, researchers may use a cell stress or viability assay.

## Resource availability

### Lead contact

Further information and requests for resources and reagents should be directed to and will be fulfilled by the lead contact, Issei S. Shimada (ishimada@med.nagoya-cu.ac.jp).

### Technical contact

Technical questions on executing this protocol should be directed to and will be answered by the technical contact, Issei S. Shimada (ishimada@med.nagoya-cu.ac.jp).

### Materials availability

All unique reagents generated in this study are available from the lead contact with a completed materials transfer agreement.

### Data and code availability

The code is shown in the code box in the “Write the program” section and in Figure 4. The code is available at Zenodo: https://zenodo.org/records/21673121.

## Acknowledgments

This work was supported by the Outstanding Research Group Support Program of 10.13039/501100008883Nagoya City University grant no. (2401101), the Grant-in-Aid for Research in Nagoya City University (2621101) and 10.13039/501100000646Japan Society for the Promotion of Science Grant-in-Aid for Scientific Research (24K02425 and 26K23473 to I.S.S. and 26K09889 to Y.K.). We thank the Research Equipment Sharing Center/Core Laboratory at Nagoya City University for technical assistance (JPMXS0441500026). We appreciate Kohki Shimada at Shioji Junior High School for his technical assistance.

## Author contributions

I.S.S. and Y.K. conceived the project, designed the experiments, analyzed most of the data, and wrote the manuscript. I.S.S. performed most of the experiments.

## Declaration of interests

The authors declare no competing interests.

## Declaration of generative AI and AI-assisted technologies in the writing process

During the preparation of this work, the authors used ChatGPT 5.6 and DeepL Write to check for grammatical errors. After using these tools, the authors reviewed and edited the content as needed and take full responsibility for the content of the publication.
